# Updated Review on the Mechanisms of Pathogenicity in *Mycobacterium abscessus*, a Rapidly Growing Emerging Pathogen

**DOI:** 10.3390/microorganisms11010090

**Published:** 2022-12-29

**Authors:** Paula López-Roa, Jaime Esteban, María-Carmen Muñoz-Egea

**Affiliations:** 1 Department of Clinical Microbiology, Hospital Universitario 12 de Octubre, 28041 Madrid, Spain; 2 Department of Clinical Microbiology, IIS-Fundación Jiménez Díaz, UAM, 28040 Madrid, Spain; 3 CIBERINFEC, CIBER de Enfermedades Infecciosas, 28029 Madrid, Spain

**Keywords:** biofilm, intracellular, antimicrobial resistance, *Mycobacterium*, *Mycobacterium abscessus*

## Abstract

In recent years, *Mycobacterium abscessus* has appeared as an emerging pathogen, with an increasing number of disease cases reported worldwide that mainly occur among patients with chronic lung diseases or impaired immune systems. The treatment of this pathogen represents a challenge due to the multi-drug-resistant nature of this species and its ability to evade most therapeutic approaches. However, although predisposing host factors for disease are well known, intrinsic pathogenicity mechanisms of this mycobacterium are still not elucidated. Like other mycobacteria, intracellular invasiveness and survival inside different cell lines are pathogenic factors related to the ability of *M. abscessus* to establish infection. Some of the molecular factors involved in this process are well-known and are present in the mycobacterial cell wall, such as trehalose-dimycolate and glycopeptidolipids. The ability to form biofilms is another pathogenic factor that is essential for the development of chronic disease and for promoting mycobacterial survival against the host immune system or different antibacterial treatments. This capability also seems to be related to glycopeptidolipids and other lipid molecules, and some studies have shown an intrinsic relationship between both pathogenic mechanisms. Antimicrobial resistance is also considered a mechanism of pathogenicity because it allows the mycobacterium to resist antimicrobial therapies and represents an advantage in polymicrobial biofilms. The recent description of hyperpathogenic strains with the potential interhuman transmission makes it necessary to increase our knowledge of pathogenic mechanisms of this species to design better therapeutic approaches to the management of these infections.

## 1. Introduction

Among the genus *Mycobacterium*, Rapidly Growing Mycobacteria (RGM) represents a group that includes roughly half of the species of the genus. Most of these organisms are acquired from environmental sources and have never been described as a cause of infection. However, non-pigmented species within this group have been described in human clinical samples for more than 100 years [1]. The improvement in the isolation and identification techniques has led to an increased awareness of the importance of RGM as human opportunistic pathogens. The species of RGM are capable of producing disease in humans consist of non-pigmented and pigmented species primarily belonging to the *M. fortuitum* group and the *M. chelonae* abscessus group [2].

The *Mycobacterium abscessus* species comprises three subspecies: *M. abscessus* subsp. *abscessus*, *M. abscessus* subsp. *bolletii*, and *M. abscessus* subsp. *Massiliense* [3]. Some authors have differentiated the three species (*M. abscessus*, *M. bolletii*, and *M. massiliense*) and include them within a complex named the *M. abscessus* complex [4]. A recent study divided the genus *Mycobacterium* into five different genera [5], but this change has been considered a source of confusion [6]. Furthermore, new studies have shown that the classical genus *Mycobacterium* must be maintained [7]. Since there is still no consensus on the nomenclature of these taxa [8], we will use the classical genus *Mycobacterium* and the subspecies nomenclature in this review.

These organisms are responsible for nosocomial skin and soft tissue infections and pulmonary infections, especially in patients with cystic fibrosis (CF) and other lung disorders [2,9]. Although this species has been traditionally considered an opportunistic pathogen, there is now compelling evidence that the *M. abscessus* clade possesses hallmark features of a true human pathogen [10]. Moreover, the widely reported increasing incidence of *M. abscessus* infections in all types of populations provides further evidence that *M. abscessus* possesses a diverse collection of virulence factors that justify its continuous evolution into a true pathogen [11]. These virulence factors enhance *M. abscessus* survival within the host: intracellular survival, enhancement of bacterial cording, biofilm development, and immune masking to escape detection [12].

Although the mechanisms of transmission are well characterized for *Mycobacterium tuberculosis*, the exact routes of transmission of *M. abscessus* are still under investigation. Recent studies have demonstrated human-to-human transmission using whole-genome sequencing (WGS). This transmission event is well documented in patients with close contact [10,12] but should be questioned when possible environmental sources of infection are found [13,14]. The mechanisms of transmission seem to help *M. abscessus* evolve from an environmental bacterium to a transmissible human pathogen. WGS has also revealed the presence of dominant *M. abscessus* clones associated with worse patient outcomes [11]. These new epidemiological insights and pathogenic findings are likely to be related; therefore, future research could open new fields of *M. abscessus* disease research.

**Figure 1:**
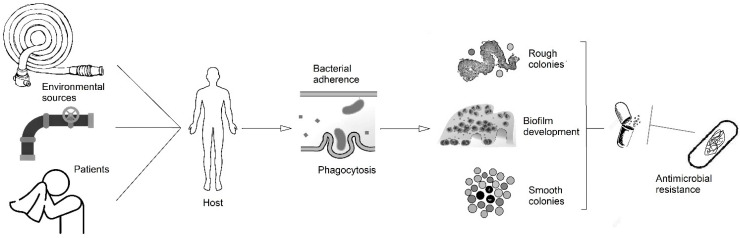
Scheme of the pathogenesis of *M. abscessus* disease from the potential sources to the development of biofilms, intracellular survival, and antimicrobial resistance.

It is necessary to understand the unique disease pathogenesis possessed by this species to reduce the global incidence of this emerging pathogen. This review aims to summarize our current knowledge of mechanisms of pathogenicity of *M. abscessus* and their implications in human disease (Figure 1).

## 2. Models of Intracellular Survival

Irrespective of being an RGM, *M. abscessus* shares some traits with *M. tuberculosis*, including the ability to survive the bactericidal response of macrophages and fibroblasts in the lungs and skin [13,15], as well as the capacity to persist silently within granulomatous structures and to produce pulmonary caseous lesions [14].

**Figure 2:**
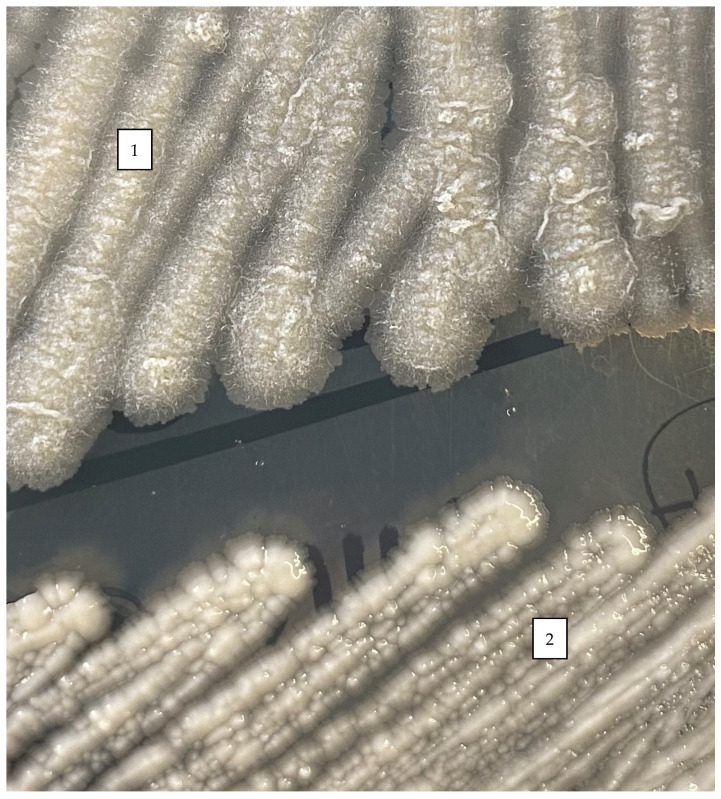
Rough (**1**) and smooth (**2**) phenotypes of colonies of *M. abscessus* in Middlebrook 7H10 agar after 5 days of incubation at 30 °C.

Like other mycobacteria, *M. abscessus* is expressed as either a smooth (S) or a rough^®^) colony morphotype (Figure 2). These morphological differences between S and R variants rely on the presence or absence of glycopeptidolipids (GPL). GPL regulate bacterial hydrophobicity and consequently biofilm formation. The pathophysiological characteristics and virulence mechanisms of the R or S variants also appear to be different [13].

Studies using cellular and animal models (mammalian and non-mammalian) have evaluated phagocytosis and intracellular survival of *M. abscessus* as pathogenic factors. *M. abscessus* has a high aggregation ability, which leads to the formation of large aggregates (clumps) that normally remain in phagocytic cups instead of being internalized [16]. As observed with other mycobacteria, R variants did not undergo degradation in phagolysosomes [17,18]. Moreover, the presence of the R variant inside macrophages induced cell apoptosis through the formation of autophagic vacuoles. It is worth highlighting that within the phagosome, the smooth and rough variants differ considerably, resulting in two phenotypically distinct infections [19]. The smooth variant is held in close apposition between the phagosomal membrane and the bacterial surface and inhibits phagosomal maturation and activation of autophagy and apoptosis-mediated pathways [20]. However, with the rough variant, the entire social phagosome rapidly fuses with the lysosome, resulting in phagosomal acidification and activation of apoptosis and autophagy [18]. This potent apoptosis-induced cell-death activity promotes extracellular replication of the R variant by rapid cord formation, preventing phagocytosis of the bacilli by neutrophils and macrophages and leading to abscess formation with tissue destruction and acute infection [21,22].

By contrast, a macrophage infected with the S form presents loner phagosomes containing one bacillus [16]. Some authors have reported that this variant is also found to reside in slightly acidified non-mature phagosomes that are not able to fuse with lysosomes resulting in a maintained intramacrophage survival of the S variant over the R variant and the increased resistance of the S variant against cellular bactericidal mechanisms [17,18]. Moreover, contrary to that described with the R morphotype, the S variant is not capable of forming cords, so it is rapidly phagocytosed [22]. As the granuloma matures, the adaptive immune response is activated, and B and T lymphocytes are recruited to coat the granuloma. The smooth variant of the *M. abscessus* may irreversibly transition to a rough variant, resulting in the formation of massive bacterial cords capable of resisting phagocytosis. The biological triggers responsible for this process are still unknown [19].

Different cell line models have been used to assess these intracellular survival properties of *M. abscessus* in vitro. In 1999, Byrd and Lyons [13] used a previously described fibroblast microcolony assay [23], trying to assess the differences between R and S colony phenotypes. According to this study, R strains grew to form elongated microcolonies inside the fibroblast cells, where cording forms could be observed, but S strains showed rounded microcolonies that appeared to be outside the fibroblasts. This study stated the ability of *M. abscessus* to grow intracellularly in non-professional phagocytes, such as fibroblasts. However, using this model, other authors have not obtained the same results with all the strains that show the R phenotype [24], so other factors must be involved in the interaction between this cell line and other mycobacteria.

Another cell line used in the same study was human monocytes [13]. In this model, again, the behavior of the two phenotypes was different. The R strains were able to form aggregates when co-cultured with this cell line, while the S strains showed no aggregates. The different behavior of the R and S phenotypes in both cell lines suggests the presence of pathogenic factors in R strains that are not present in S ones.

More recently, Roux et al. [18] used a macrophage cell culture model and showed that R *M. abscessus* cells were able to multiply inside the cells, while S cells were not. This model has been used more recently with other species, showing that the R strains of both *M. abscessus* and other RGM can survive inside the macrophages more easily than the S strains [25].

Another different model was described by Ganbat et al. [26]. The model is an in vitro study that uses lung tissue instead specific cell lines. This model allows the study of the interaction of mycobacteria with many different cell lines in the same study and showed that mycobacteria can infect all the cells in the model (pneumocytes and immune cells), with differences between species. In the study, the different species (including *M. abscessus*) showed different properties regarding the different damage to each cell type present in the tissue. The authors also showed the differences between S and R strains in different aspects of cell internalization and survival, showing the possibilities of this model for studying the interactions between mycobacteria and the different cell lines present in the actual lung tissue.

Regarding experimental models, although these models are still used [27], they remain costly and time-consuming in addition to present ethical and budgetary hurdles associated with the use of mammals [28]. Moreover, studies evaluating murine models described a limited ability to induce chronic infection that simulates pulmonary infection in humans [29]. Therefore, alternative non-mammalian models, such as *Galleria mellonella* moth larvae, *Drosophila melanogaster,* and zebrafish embryos have been developed to study both host and mycobacterial determinants of pathogenesis during chronic infection with *M. abscessus* [25,29,30].

Zebrafish is a model that can be chronically infected with *M. abscessus*. The optical transparency of embryos has been used to visualize the formation of extracellular cords by the R variant in vivo. Using this in vivo model, some authors [31,32] described the ability of both variants to induce granulomas and confirmed the hypervirulence of the R morphotype related to the massive production of extracellular cords. As observed in these studies on the zebrafish infection model, *M. abscessus* can irreversibly switch from the smooth to the rough variant during persistent infection, resulting in granuloma rupture and bacterial cord formation [19,31].

*Drosophila melanogaster* has become a well-established model for the study of innate immunity and is increasingly being used as a tool to study host–pathogen interactions [33]. *Drosophila melanogaster* is a genetically tractable model host for *M. abscessus*. The *M. abscessus* infection results in dissemination in the fly body, followed by death, which is accompanied by severe indirect damage to the flight muscle and brain. *M. abscessus* can grow and replicate in *D. melanogaster* and elicits a humoral immune response [30]. All of these studies have identified host processes and factors required for cellular entry, to control or resist nonpathogenic mycobacteria, or factors that are deregulated as a result of mycobacterial infection [30,33].

Another invertebrate model, Galleria mellonella, is a reproducible, low-cost, and ethically acceptable in vivo model that is increasingly recognized as an alternative to studying microbial infections [25,28]. This model has been used to study the pathogenicity and virulence of *M. tuberculosis* and other NTM infections. Studies of the *M. abscessus* infection model in *Galleria mellonella* reported that *M. abscessus* replicated and induced granuloma-like responses in infected larvae, leading to larval mortality [25,29]. Furthermore, according to the studies using cell lines discussed above, larvae infected with the R variant of *M. abscessus* had a lower survival rate than those infected with the smooth one.

## 3. Models of Biofilms

A key strategy of NTM pathogenesis is the ability to form biofilms. Biofilms are suspected to play an important role in *M. abscessus* infections [9]. They are aggregates of bacteria that are established in response to stress. As part of this survival strategy, the bacteria within the biofilm undergo genetic and metabolic changes [34].

There is evidence of the formation of biofilm-like microcolonies in the lungs [35]. In patients with pre-existing lung disease, *M. abscessus* can first develop a biofilm, colonizing the host, and subsequently progress to invasive disease [36]. It should be noted that mycobacteria growing inside biofilms become tolerant to antibiotics, contributing to their drug resistance. In fact, in vitro biofilm models of *M. abscessus* have been shown to have decreased susceptibility to several first-line antibiotics, such as cefoxitin, amikacin, and clarithromycin [37,38].

**Figure 3:**
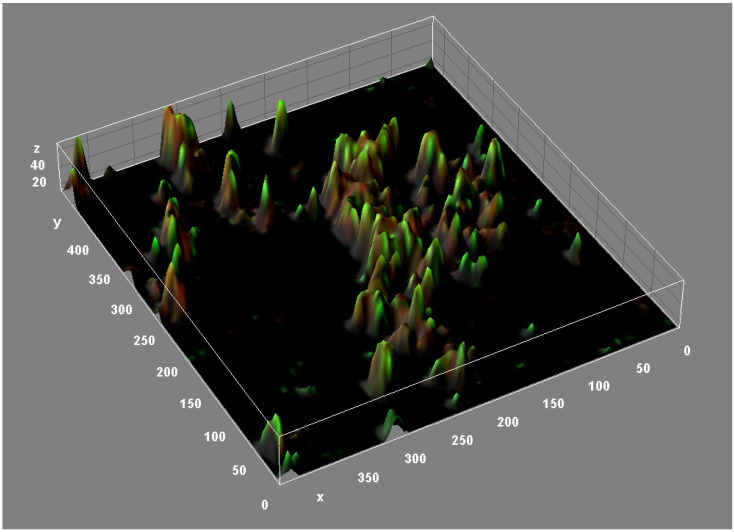
Three-dimensional reconstruction of an *M. abscessus* biofilm (CLSM, Backlight live-dead stain).

The structure of *M. abscessus* biofilms is highly complex. Confocal microscopy (Figure 3) and other studies have revealed the presence of lipids, proteins, carbohydrates, and extracellular DNA (eDNA) in the *M. abscessus* biofilm matrix [39]. These structural components appear to play a crucial role in the formation and maturation of biofilms of different bacterial species [40]. Lipids, particularly mycolyl-diacylglycerol, mycolic acids, and glycopeptidolipids, are essential for biofilm formation. In fact, strains defective in cell wall lipid export are defective in biofilm formation [39,41]. DNA is abundant in *M. abscessus* biofilms (relative biovolume = 0.846) and dispersed throughout the biofilm, especially in areas with low cell counts [39]. eDNA plays a key structural role in promoting adhesion to surfaces and facilitating bacterial aggregation of the biofilm [42,43]. In contrast to the important role played by carbohydrates in *M. chelonae* and *M. tuberculosis* biofilms [44,45,46], carbohydrates appear dispersed in *M. abscessus* biofilms, as evidenced by the presence of trace amounts in the extracellular matrix [39].

Importantly, the mycolic acids of *M. abscessus* biofilms undergo specific traceable molecular changes, as demonstrated by a recent study using transcriptomic analysis [39]. This study revealed the up-regulation of pathways involved in glyoxylate derivatization, redox metabolism, and mycolic acid biosynthesis. Genes involved in mycolic acid elongation and desaturation were highly up-regulated in *M. abscessus* biofilms, and, reflecting these findings, biochemical analysis of mycolates revealed molecular changes and an increase in mycolic acid chain length [39].

No clear differences have been described between environmental and clinically relevant isolates regarding colony morphotype. While one study showed no differences between strains that colonize the respiratory tract and those that can be considered as causing infection [47] regarding the colony morphotype, there are no references regarding this issue. However, because rough variants are considered more pathogenic, it seems logical that smooth colonies appear more frequently among environmental isolates, especially because these strains are usually strong biofilm producers.

Pulmonary colonization by *M. abscessus* begins with smooth strains producing abundant GPL (minimal dimycolate trehalose) and robust biofilm. However, in invasive infection, rough strains have genetic lesions at the GPL loci and are responsible for the production of higher levels of trehalose dimycolate and, consequently, for the formation of massive bacterial cords [48].

Biofilms formed by R colony types have a higher degree of mechanical resistance compared to biofilms formed by S colony types [49]. It is noteworthy that a predominant paradigm of chronic *M. abscessus* infection is that the S morphotype is a non-invasive, biofilm-forming, persistent phenotype, and the R morphotype is an invasive phenotype incapable of forming biofilms. R variants of *M. abscessus* also form pellicular biofilms [39]. A recent study has shown that the rough morphotype is hyperaggregative and forms biofilm-like aggregates, which, like the biofilm aggregates of the S variant, are significantly more tolerant than the planktonic variants to acidic pH, hydrogen peroxide, and treatment with amikacin or azithromycin [50].

*M. abscessus* can live in the same ecosystems as *Pseudomonas aeruginosa* and *Burkholderia cepacia*, and patients can be infected from the same microbial reservoir [51]. *P. aeruginosa* is the most prevalent species in CF patients [52]. However, the microbiota of the pulmonary ecosystem may also be composed of other microorganisms, including NTM, which are mostly acquired from environmental reservoirs. Then, presumably, bacteria become established in the lungs of CF patients forming polymicrobial biofilms [53].

CF lung microbial communities encounter frequent antibiotic therapy, host immune factors, and an altered lung environment [53]. In vitro conditions that mimic the airways of CF patients appear to facilitate the establishment of *M. abscessus* infection, and the removal of magnesium from the environment may affect the ability of the pathogen to establish infection [54]. Moreover, some studies showed that *P. aeruginosa* inhibited *M. abscessus* biofilm formation under control conditions and that antimicrobial treatment selectively targeting *P. aeruginosa* decreased this competitive interaction, thereby increasing *M. abscessus* survival [55].

## 4. Molecular Mechanisms

*M. abscessus* possesses a special cell wall that contributes greatly to the high level of antibiotic resistance and pathogenicity. It is composed of complex lipids, including glycopeptidolipids (GPL), which serve several important functions. They are involved in gliding motility, biofilm formation, interaction with host cells, and intramacrophage trafficking [56]. The R form has been shown to lack GPL and causes more severe infections in mice by strongly inducing TNF secretion by macrophages [57]. Describing the molecular mechanisms that allow *M. abscessus* to switch from a smooth to a rough variant is important to understand the role and contribution of GPL in virulence and pathogenicity. Comparative genomic studies of several smooth and rough isogenic pairs identified several genetic changes, such as small insertions (such as nucleotide insertions in mps1) or deletions (such as single-nucleotide deletions in mmpL4b) and single-nucleotide polymorphisms in the GPL cluster, showing that the transition to a rough morphotype is irreversible [58]. However, the mechanisms that regulate GPL expression have not yet been described. Lsr2 is a small nucleoid-associated protein that is highly conserved in mycobacteria, including *M. abscessus,* and some studies have observed that, in the rough variant of *M. abscessus*, there is a higher expression of Lsr2 than in the smooth variant [59].

The high antibiotic resistance of *M. abscessus* is also because its genome encodes many proteins potentially involved in drug efflux systems, including members of the major facilitator family, the ATP-binding cassette transporters (ABC transporters), and Mycobacterial membrane large proteins (MmpL proteins) [60]. *M. abscessus* contains an mgtC gene, which is important for the intracellular lifestyle. MgtC is a known virulence factor involved in intramacrophage survival and adaptation to Mg(2+) deprivation. A study in mice demonstrated the inhibition of MgtC in vivo by immunization with *M. abscessus* MgtC DNA, which exerted a protective effect against an aerosolized *M. abscessus* challenge in FVB mice (ΔF508). Immunization with formulated DNA was likely associated with the production of specific antibodies against MgtC, which may have stimulated a protective effect by counteracting MgtC activity during *M. abscessus* infection. These results underline the importance of MgtC of *M. abscessus* in vivo and provide a basis for the development of new therapeutic tools against *M. abscessus* lung infections in CF patients [59].

In addition, several studies have shown that Lsr2, a nucleoid-associated protein (NAP) that has been found strictly in actinobacteria, including mycobacteria, is required for the survival of *M. abscessus* in zebrafish and mice. Lsr2 plays a critical role in the virulence of *M. abscessus* in different animal hosts and highlights the requirement of Lsr2 for persistence in mice, particularly in the lungs, which represents the main target organ during infection in patients, especially in those with already underlying lung disease [31,59]. The absence of lsr2 in *M. abscessus* has been shown to cause decreased virulence in cell and animal models [59].

Part of the infective capacity of *M. abscessus* could be related to the presence of selected virulence genes of non-mycobacterial origin in its genome that were originally acquired by ancient Horizontal Gene Transfer (HGT) or lateral gene transfer events from unrelated microorganisms [60]. Analysis of the complete genome sequence of *M. abscessus* revealed the presence of many specific genes in common with two pathogens most frequently isolated from cystic fibrosis (CF) patients: *P. aeruginosa* and *B. cepacia*. Presumably, these genes were acquired from distantly related environmental bacteria through HGT [60].

Most of the small number of protein families found overrepresented in *M. abscessus* are known to be associated with mycobacterial pathogenicity (e.g., PE and PPE proteins (two large families of proteins typical of mycobacteria), mammalian cell entry (MCE), YrbE proteins, LpqH lipoprotein precursors (an immunomodulatory surface lipoprotein of *M. tuberculosis*), and lipases/esterases/monooxygenases) [60]. Other key virulence factors appear to have been acquired horizontally from distantly related environmental bacteria with a high G+C content, mainly actinobacteria and pseudomonads, e.g., phospholipase C, MgtC, MsrA and Fe(3+) ABC transporter [60]. Others, such as members of the Arsenate Reductase (ArsC) family, salicylate hydroxylases, and cysteine desulfurases, are characteristic of soil- or water-dwelling organisms [60].

MCE and YrbE proteins allow mycobacteria to invade host cells. There are seven MCE operons in *M. abscessus* [60]. In other microorganisms such as actinomycetes, it has been shown that the number of MCE operons can be related to pathogenicity [61].

Several genes have been identified that are important for various aspects of *M. tuberculosis* pathogenicity, including those encoding 19-kDa proteins that induce macrophage apoptosis, and the presence of multiple sigma factors that contribute to mycobacterial adaptation and survival [12]. *M. abscessus* shares some of these virulence factors described in *M. tuberculosis*, which are described below. LpqH-like proteins LpqH, also known as 19 kDa protein, is an immunodominant antigen recognized by T lymphocytes and sera from tuberculosis patients [62]. *M. abscessus* possesses four genes encoding LpqH-like proteins scattered throughout the genome, suggesting that these molecules may be involved in the pathogenicity of *M. abscessus*, possibly through modification of the host response [60]. In addition, *M. abscessus* has virulence factors homologous to the five sigma factors shown to be involved in *M. tuberculosis* virulence (SigA, SigC, SigD, SigE, SigH) and also has a protein homologous to the virulence transcription factor VirS of *M. tuberculosis* [60].

There is a set of genes essential for the intracellular survival of *M. abscessus* within amoebae and macrophages. Bacterial phospholipase Cs are key virulence factors that allow intracellular pathogens to escape from phagosomal vacuoles by disrupting eukaryotic membranes [63]. Phospholipase C from *M. abscessus* closely resembles proteins from *Streptomyces* sp., *Chromobacterium violaceum,* and *P. aeruginosa* [60]. *M. abscessus* also contains an MgtC gene that is important for the intracellular lifestyle. The acquisition of MgtC genes by HGT is common among microbes and has been associated with pathogenicity [64]. The MgtC gene in *M. abscessus* appears to have been acquired by HGT, probably from actinobacteria [64]. The strong induction of MgtC has been demonstrated in *M. abscessus* at both the transcriptional and translational levels when bacteria reside within macrophages or after Mg(2+) deprivation. In addition, it was shown that MgtC from *M. abscessus* was recognized by sera from *M. abscessus*-infected CF patients [65].

Free-living amoebae are thought to represent an environmental niche in which amoeba-resistant bacteria may evolve toward pathogenicity [66]. Many species of mycobacteria, including *M. abscessus,* can survive in amoeba trophozoites and in the later stages of cysts [67]. Whether it is a potential host is not yet known. Survival within amoebae may be an intermediate stage of the life cycle that allows *M. abscessus* to persist in the environment in a protected niche while preparing the bacterium for the colonization of other hosts [68].

A recent study has identified by genomic analysis several specific genetic elements that may promote the intracellular life of *M. abscessus*, particularly within amoebae [19]. *M. abscessus* contains several genes that are characteristically found only in pathogenic bacteria. One of these is MAB_0555, which encodes a putative phospholipase C (PLC) that is absent in most other fast-growing mycobacteria. Furthermore, it has been shown through a mutant of *M. abscessus* that the loss of PLC activity is deleterious to the intracellular survival of *M. abscessus* in amoebae [69].

Due to the ability of *M. abscessus* to survive and replicate within the free-living amoeba, an essential role of the type VII secretion system (T7SS) of *M. abscessus* was discovered, corroborating that a genetic factor may have naturally selected for intracellular survival of *M. abscessus* [69]. ESX-4 in *M. abscessus* is known to block phagosomal acidification and disrupt phagosomes, similar to the role of ESX-1 in *M. tuberculosis* [69]. In humans and other animal species, transcriptomic changes in *M. abscessus* during intracellular growth in macrophages have demonstrated the up-regulation of genes such as heat shock and oxidative stress genes (e.g., GroEL-ES, a molecular chaperone complex, and hsp) to cope with intracellular stress [68].

## 5. Mechanisms of Antimicrobial Resistance

Infections due to *M. abscessus* are difficult to treat because this mycobacterium is intrinsically resistant not only to the classical anti-tuberculous drugs but also to most of the antibiotics that are currently available, including macrolides, aminoglycosides, rifamycins, tetracyclines, and β-lactams [70,71]. Intrinsic resistance is attributed to many resistance mechanisms, including the low permeability of the cell envelope, the induction of drug efflux pumps, and numerous enzymes that can modify either the drug target or the drug itself [19]. To date, acquired resistance has only been reported for aminoglycosides and macrolides, which rely on modifications of the genes encoding the antibiotic targets (*rrs* and *rrl*, respectively) [72].

### 5.1. Intrinsic Resistance

The low permeability of the mycobacterial cell envelope plays an essential role in the natural resistance of *M. abscessus* to antibiotics. The high lipid content (up to 60% of the dry weight of the bacteria) and unusual thickness of the mycobacterial cell wall are considered the main factors contributing to this low permeability and provide an effective barrier for hydrophilic and lipophilic agents [10].

Nevertheless, the cell wall barrier alone cannot explain all of the intrinsic drug resistance seen in *M. abscessus*, and other factors appear to be involved. It is well documented that the cell wall also contains porins that allow the diffusion of potentially lethal amounts of compounds and hydrophilic antibiotics through the envelope [73,74]. Once internalized, the antibiotics can reach their target in the cytoplasm and activate the expression of potential drug-resistance genes. Moreover, the cell envelope, particularly the porins, can act synergistically with antibiotic-inducible internal systems to compete with the effects of drugs [73]. This internal system includes efflux pumps, antibiotic-modifying/inactivating enzymes, target-modifying enzymes, and genes conferring metal resistance.

Active efflux pumps represent one of the most important causative mechanisms of antibiotic resistance in mycobacteria [75]. They protect bacteria against toxic molecules and promote cell homeostasis by exporting toxins or metabolites to the extracellular environment. *M. abscessus* encodes protein members of the major facilitator family ATP-binding cassette (ABC) transporters, as well as mycobacterial membrane protein large (MmpL) families [60]. The MmpL transporter family is a subclass of a large family of multi-drug-resistance pumps known as Resistance-Nodulation-Cell-Division (RNCD) permeases. MmpL is involved in lipid transport and other compounds across the cell envelope of mycobacteria [76]. Although there is evidence that MmpL7 in MTB plays a role in intrinsic drug resistance [11], the role of this family in *M. abscessus* antibiotic resistance is not well understood [10]. Another group of efflux pumps is the ABC-type multidrug transporters, which use ATP energy to pump molecules across membranes [10]. They can be categorized either as exporters (which remove substrates to the external environment) or as importers (which uptake extracellular molecules) [71,77].

Macrolide antibiotics are widely used to treat infections caused by NTM and are one of the mainstays of *M. abscessus* treatment. However, *M. abscessus* tends to respond poorly to macrolide chemotherapy, even when they appear sensitive to clarithromycin in vitro [13]. The *erm*(41) gene is the main mechanism of innate *M. abscessus* macrolide resistance. The functionality of the *erm*(41) gene differs depending on the subspecies. Most remarkably, *M. abscessus* subsp. *massiliense* harbours a large deletion in the *erm*(41) gene, rendering the gene non-functional, and so the bacterium is susceptible to macrolides [78]. In *M. abscessus* subsp. *bolletii* and *M. abscessus* subsp. *abscessus,* a T/C polymorphism at position 28 of the *erm*(41) sequence determines the appearance of inducible macrolide resistance. However, only those isolates that harbor a T28 *erm*(41) sequevar develop inducible resistance [79].

*M. abscessus* also produces enzymes that potentially degrade or modify antibiotics, which can result in their inactivation and thereby contribute to resistance to most classes of antibiotics. Phosphotransferases and acetyltransferases mediate the susceptibility to aminoglycoside antibiotics. The best-characterized aminoglycoside-modifying enzyme in *M. abscessus* is aminoglycoside N-acetyltransferase (AAC 2′) [72].

Constitutive β-lactamase production contributes to β-lactam resistance in *M. abscessus* by reducing the effective concentration of β-lactams at the site of action. *M. abscessus* possess an endogenous β-lactamase (BlaMab) encoded by MAB2875 [80]. This enzyme presents a broad spectrum activity and can effectively hydrolyze several members of first- and second-generation cephalosporins, carbapenems, and penams [81]. Although cefoxitin and imipenem are substrates of BlaMab, they are hydrolyzed at a very slow rate, which can explain the moderate activity of these drugs against *M. abscessus* [19].

Rifampicin is a rifamycin used as a first-line drug in the treatment of tuberculosis; however, these drugs hardly exhibit any activity against *M. abscessus*. Recently, genetic studies revealed the existence of a MAB0591-encoded ADP-ribosyltransferase as the major determinant for high levels of intrinsic rifamycin resistance in this pathogen [82,83].

The presence of variant nucleotides within conserved genes targeted by drugs has been associated with ethambutol and fluoroquinolone resistance [71]. *M. abscessus* exhibits intrinsic high-level resistance to ethambutol mostly due to the presence of variant nucleotides within the conserved embB ethambutol resistance-determining region (ERDR) [84]. Fluoroquinolone-resistant isolates of *M. abscessus* present an amino acid substitution at position 83 (Ser83Ala) in the quinolone-resistance-determining-region (QRDR) of *gyr*A [10,85].

Moreover, apart from antibiotic-specific internal drug resistance mechanisms, *M. abscessus* is equipped with a family of transcriptional regulators potentially involved in conferring drug resistance (the whiB gene family) [86]. MTB WhiB7 is involved in the regulation of significant cellular processes related to drug resistance. Previous studies have shown that *M. abscessus* whiB7, a homolog of MTB whiB7, has an important role in the intrinsic resistance of *M. abscessus* to several ribosome-targeting antibiotics [87].

### 5.2. Acquired Resistance

*M. abscessus* strains can acquire aminoglycoside and macrolide resistance due to extensive, repeated, or inappropriate use of these antibiotics, which inhibit protein biosynthesis by binding to the small and large ribosomal subunits, respectively [83]. Acquired resistance to aminoglycosides is related to mutations in *rrs*, the 16S rRNA gene. In particular, the A1408G substitution in *rrs* induces high levels of resistance to kanamycin, amikacin, and tobramycin [71,88].

Point mutations in a region of the *rrl* gene encoding the peptidyltransferase domain of 23S rRNA confer acquired resistance to macrolides. The main molecular mechanism of clarithromycin-acquired resistance reportedly occurs through adenine point mutations at either position 2058 (A2058G) or position A2059 in the 23S rRNA gene [89].

To conclude, *M. abscessus* has become the most virulent and antibiotic-resistant member of the RGM group. The development of molecular methods to study *M. abscessus* antibiotic resistance will improve our understanding of the mechanisms responsible for treatment failure of infection due to this pathogen. In addition, since *M. abscessus* subsp. *massiliense* appears more susceptible than *M. abscessus* subsp. *abscessus* and *M. abscessus* subsp. *bolletii,* the molecular identification within the complex might also improve the treatment of these infections. Microbiology laboratories should be aware that phenotypic detection of inducible macrolide resistance requires extended incubation (14 days).

## 6. Clinical Implications

**Figure 4:**
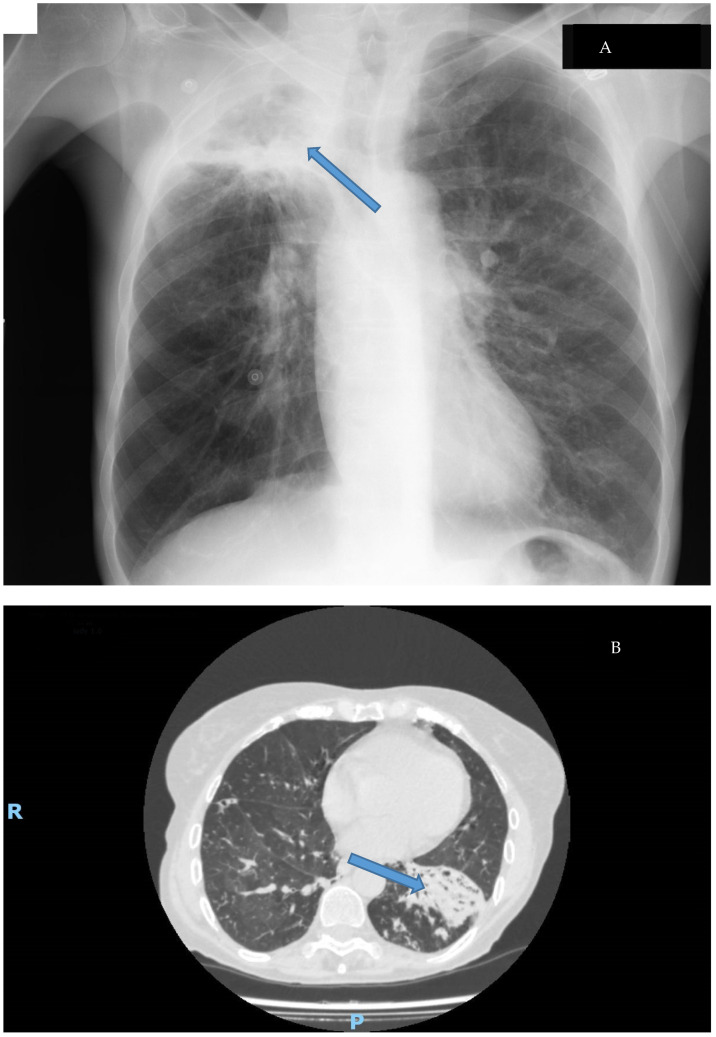
Types of lung disease caused by *M. abscessus*: (**A**): Cavitary disease (cavitation in the upper lobe of the right lung Thoracic X-ray); (**B**): Fibronodular disease (alveolar infiltrates with bronchiectasis in CT-Scan). Disease is signaled by an arrow.

Clinical disease caused by *M. abscessus* shows a similar pattern to other NTMs. These microorganisms usually cause many different syndromes, some of them related to foreign bodies, and *M. abscessus* is no different. The most common disease caused by this mycobacterium is a respiratory disease, usually showing two different syndromes (Figure 4): cavitary disease, which usually appears in patients with preexisting anatomic conditions (such as bullae or old tuberculosis cavitations), and fibronodular disease, which also emerges in patients with underlying conditions such as chronic bronchiectasis or cystic fibrosis [9,10,53,90]. The importance of the growing number of patients with these diseases and the multi-drug-resistant nature of this pathogen has motivated the development of specific treatment guidelines for these patients. [91,92].

However, *M. abscessus* can also cause other diseases in humans, mostly related to different procedures, such as surgery or aesthetic procedures [93,94]. Some of these cases have appeared as outbreaks, sometimes related to contaminated environmental sources [95,96,97,98]. The introduction of molecular epidemiology tools has been of great importance for the characterization of these outbreaks because the phenotypic characteristics of this mycobacterium are so common that strain differentiation must be carried out by molecular analysis [11,97,99,100].

The implications of the above-cited pathogenic factors have been well known for many years. Just as *M. tuberculosis* is an extraordinary paradigm of intracellular pathogens that do not have classical virulence factors, intracellular survival has also been considered the key pathogenic factor among other mycobacterial diseases. According to the latest theories about the infection process, during the first steps of infection caused by *M. abscessus*, the mycobacteria are phagocytized and can survive inside the phagocytes, inducing the formation of a granuloma (Figure 1). Inside this structure, the mycobacteria can change their properties, evolving into an R phenotype that destroys the cells and can cause disease [19]. The ability to survive inside the macrophages and other phagocytic cells is considered not only one of the ways to evade the immune system during the early stages of the infection but also a survival mechanism inside amoebae in the environment, which constitute a source of infection [19,101]. This ability also determines the selection of antibiotic treatments, since these must be able to penetrate inside the cells to be effective against mycobacteria [91], a fact that limits the number of available drugs.

Biofilm development also has important implications in clinical disease. In the first steps of the lifecycle of the mycobacteria, these organisms are part of different polymicrobial biofilms that can include not only bacteria but also free-living amoebae. Mycobacteria can reach the host as aggregates in aerosolized samples and begin to develop new biofilms in patients [19] (Figure 1). The presence of NTM in environmental biofilms is a well-known fact and is considered the source of infection in most cases, even in outbreaks [102]. Some experiments have shown the ability of *M. abscessus* to attach to plumbing systems [103], and also the capability of this organism to be aerosolized by common activities such as the use of humidifiers [104]. Again, biofilms are involved in these situations in environmental sources that can be the origin of different diseases.

Biofilm has been considered a key pathogenic factor in many different diseases, both chronic and implant-associated [105,106], with important implications for the management of patients. In fact, recent reports have shown the presence of biofilm in tissue from patients with different types (Figure 4) of chronic lung disease [35,36]. This structure allows *M. abscessus* to increase its resistance against the immune system and antibiotics. In this sense, in vitro experiments have shown that antibiotic concentrations need to be increased 1000-fold for the eradication of the biofilm [107]. If we consider that biofilms can develop in the bronchial tree and are difficult for systemic antibiotics to reach, the treatment of these patients is an enormous challenge. Moreover, in biomaterial-related infections, surgical removal of the implant is mandatory to cure the patient. To overcome these problems, new therapeutic approaches are being studied, and some of them will likely be available shortly [108,109].

In addition to the factors mentioned above, the multirresistant nature of this mycobacterium should be considered. The importance of the resistance mechanisms that appear in *M. abscessus* limit the number of available antibiotics for the management of the diseases caused by this species. The ability to develop resistance to multiple antibiotics through the acquisition of new resistance mechanisms makes it necessary to use combined treatment schemes [92]. This fact, together with the long duration of treatment and the risk of developing unwanted adverse effects, makes the treatment of this disease a challenge for modern medicine. [10,71,92,108].

Moreover, the possibility of dual biofilms (and probably polymicrobial ones) that include *M. abscessus* [55,110,111] represents a new challenge for the management of these patients. The relationships between the different species are not well understood yet, but the implications of this fact in the clinical management of patients could be important, and the diagnosis and antibiotic selection should consider this possibility.

## 7. Conclusions

*M. abscessus* is an emerging pathogen among NTMs. Since the number of patients with diseases caused by this organism is increasing, its consideration as a true pathogen instead of an opportunistic mycobacterium is gaining consideration in modern medicine. The number of patients with underlying diseases that can be considered as risk factors is also increasing, so it is reasonably expected that the number of *M. abscessus* infections will increase during the upcoming years.

Despite these facts, the current knowledge about the pathogenic mechanisms of this mycobacterium is only shallow and mainly based on the knowledge of the pathogenic mechanisms of *M. tuberculosis*. Intracellular survival is one of the well-known mechanisms in this species and is considered an important mechanism also for *M. abscessus*. Biofilm development, another important pathogenic mechanism among mycobacteria, is also important in the pathogeny of *M. abscessus* disease. Both mechanisms have important implications in the management of patients, since they limit the number of available drugs and, therefore, make it difficult to select an effective treatment. The multi-drug-resistant nature of this species makes it even more difficult to choose the most suitable treatment for these infections. Therefore, the development of new antibiotics and new therapeutic approaches is necessary to improve the management of these patients. New studies that shed light on the pathogenesis of these infections will be extremely helpful in the development of new strategies against infection caused by *M. abscessus*.
